# Internuclear ophthalmoplegia as a presentation of procedural stroke: a case report

**DOI:** 10.1186/s13256-024-04401-w

**Published:** 2024-02-07

**Authors:** Norachai Sirisreetreerux, Krongkamol Ponglikitmongkol

**Affiliations:** 1 Cardiology Center, Chulabhorn Hospital, Chulabhorn Royal Academy, 906 Kamphaengphet 6 Road, Talat Bang Khen, Lak Si, Bangkok, 10210 Thailand; 2 Department of Medicine, Chulabhorn Hospital, Chulabhorn Royal Academy, 906 Kamphaengphet 6 Road, Talat Bang Khen, Lak Si, Bangkok, 10210 Thailand

**Keywords:** Case report, Coronary angiography, Internuclear ophthalmoplegia, Magnetic resonance imaging, Procedural stroke

## Abstract

**Background:**

Cardiac catheterization and endovascular procedures are extensively used in modern medicine, and procedural stroke is one of the major complications that the catheterization laboratory team may face in their everyday work. Recognizing the signs and symptoms of procedural stroke is crucial to ensuring appropriate management. We herein report a case of internuclear ophthalmoplegia that caused blurred vision, diplopia, and dizziness on lateral gaze as an unusual presentation of procedural stroke.

**Case presentation:**

A 60-year-old Thai woman underwent right partial colectomy and was diagnosed with stage IV diffuse large B-cell lymphoma. Pre-chemotherapy echocardiography revealed mild left ventricular systolic dysfunction, and she therefore underwent diagnostic catheterization. Coronary angiography revealed normal coronary arteries, leading to a diagnosis of non-ischemic cardiomyopathy. After the procedure, she immediately developed dizziness and diplopia. During the right lateral gaze, she exhibited impaired adduction of the left eye and horizontal nystagmus of the right eye. A diagnosis of left internuclear ophthalmoplegia was made. Magnetic resonance imaging revealed a tiny area exhibiting characteristics of an acute infarct in the left paramedian midbrain, including the left medial longitudinal fasciculus, which explained the clinical picture. Another region of restricted diffusion indicating an acute infarct was detected in the right inferior cerebellar hemisphere. Magnetic resonance angiography revealed no significant cerebral artery disease. The patient achieved full neurological recovery 6 weeks after symptom onset.

**Conclusion:**

This report describes an uncommon presentation of procedural stroke that is likely to be misdiagnosed, especially by medical staff unfamiliar with internuclear ophthalmoplegia. Despite the good prognosis of internuclear ophthalmoplegia, appropriate stroke care is crucial in patients with procedural stroke because of the risk of multiple brain infarcts.

## Background

Cardiac catheterization and endovascular procedures are widely used in modern medicine, with more than 1 million procedures being performed in the USA annually [1]. With the rapid increase in the total number of procedures, the risk of complications during and after the procedure is also growing. One such complication is procedural stroke. Despite years of progress in terms of technology, skill, and experience, the incidence of procedural stroke remains high [2–6]. According to the “8 Ds” of stroke care, the first step in appropriate management is to recognize the signs and symptoms of stroke [7]. This is especially important in cases of procedural stroke in which a medical team has limited experience with this complication. We herein describe a patient who developed procedural stroke with an unusual presentation of internuclear ophthalmoplegia (INO) that caused blurred vision, diplopia, and dizziness on lateral gaze [8].

## Case presentation

**Fig. 1:**
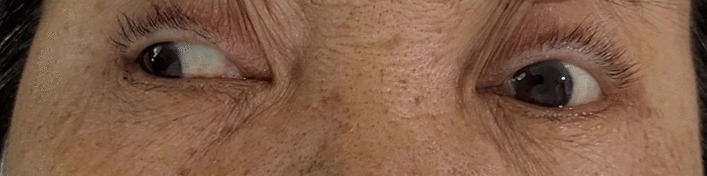
Clinical photograph showing impaired left-eye adduction during the right lateral gaze

A 60-year-old female Thai farmer was scheduled for pre-chemotherapy echocardiography after undergoing a right partial colectomy and being diagnosed with stage IV diffuse large B-cell lymphoma. Echocardiography revealed diminished left ventricular systolic function, calculated at 45% by Simpson’s method. She had type 2 diabetes mellitus, hypertension, and dyslipidemia, all of which were adequately controlled. Her medication prior to diagnostic catheterization included aspirin 81 mg/day and clopidogrel 75 mg/day for upfront medication in case of intervention needed; metformin 2000 mg/day and glipizide 10 mg/day for glycemic control; gemfibrozil 600 mg/day and atorvastatin 40 mg/day for lipid control; and enalapril 5 mg/day for antihypertensive. She never smokes but does occasionally consume alcohol. She is the mother of two children without a history of miscarriage. Her other past history was otherwise unremarkable. On her admission date, her blood pressure was 119/57 mmHg and her pulse rate was 86 beats per minute. Blood tests showed the following: fasting blood glucose, 138 mg/dL; glycated hemoglobin, 5.7%; total cholesterol, 84 mg/dL; and low-density lipoprotein, 28 mg/dL. Coronary angiography revealed normal coronary arteries, leading to a diagnosis of non-ischemic cardiomyopathy. After the procedure, she immediately experienced dizziness and diplopia. Her physical examination immediately after the procedure showed blood pressure of 140/69 mmHg and pulse rate of 86 beats per minute. Her neurological examination during the right lateral gaze showed impaired adduction of the left eye and horizontal nystagmus of the right eye. A diagnosis of left INO was made by a neurologist (Fig. 1).

**Fig. 2:**
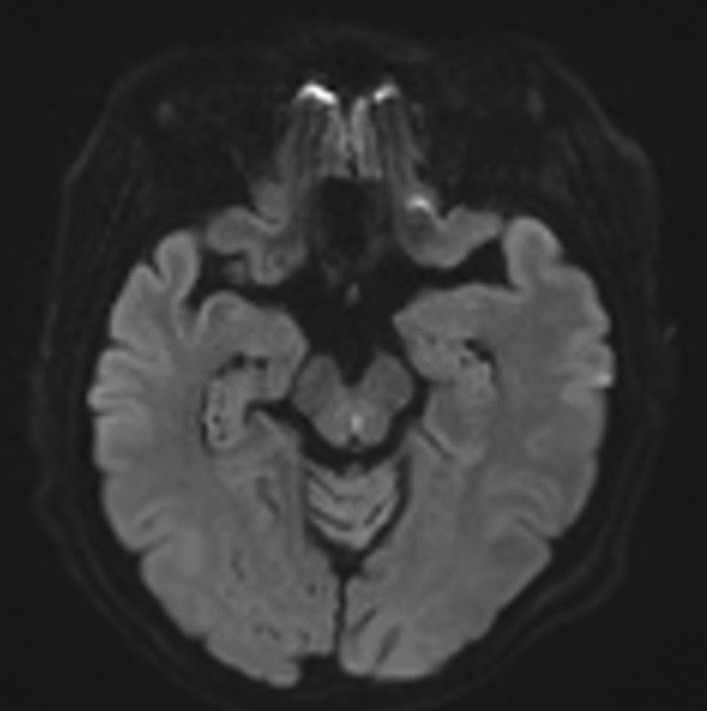
Axial diffusion-weighted image showing a diffusion defect in the left paramedian midbrain

**Fig. 3:**
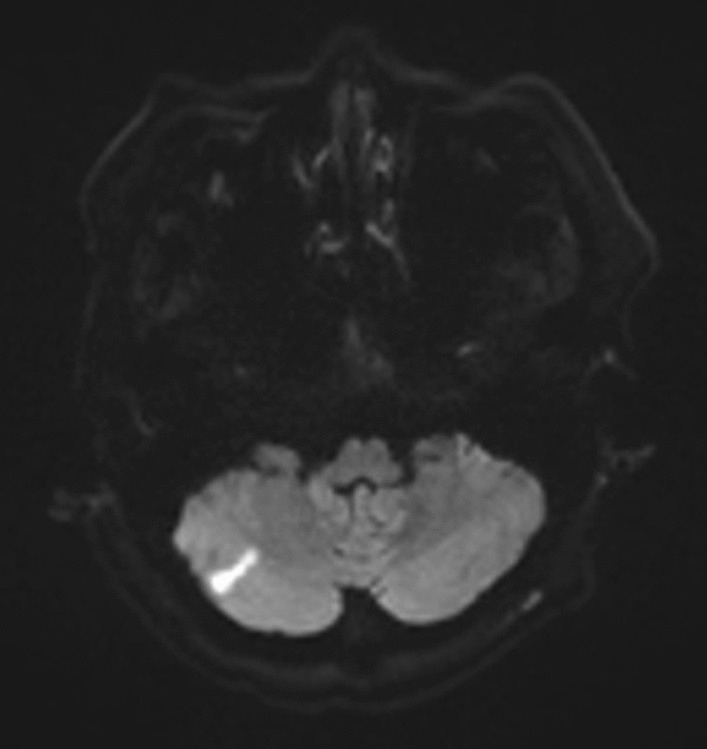
Axial diffusion-weighted image showing a diffusion defect in the right cerebellum

**Fig. 4:**
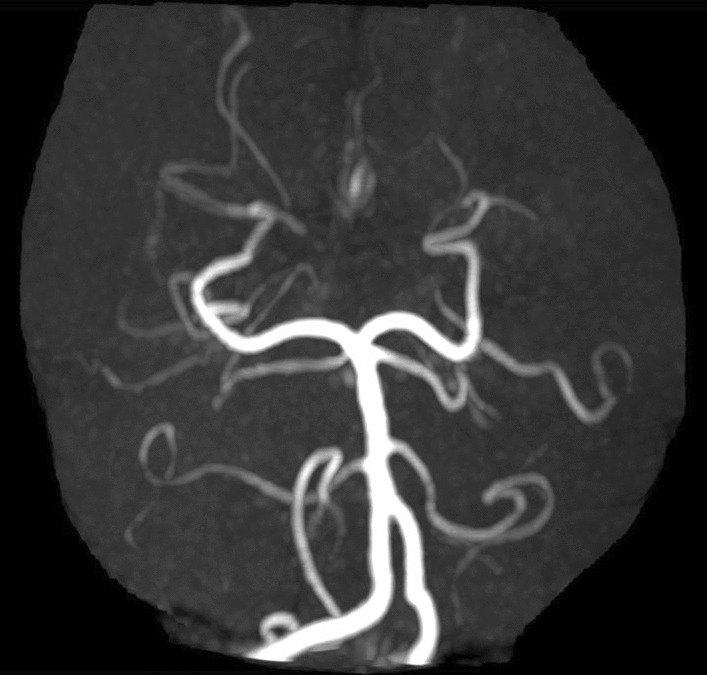
Magnetic resonance angiography showing no significant evidence of atherosclerotic disease

Immediate non-contrast computed tomography (CT) of the brain showed no signs of bleeding. Magnetic resonance imaging (MRI) on the fifth day after clinical onset revealed a tiny area exhibiting characteristics of an acute infarct in the left paramedian midbrain, including the left medial longitudinal fasciculus, consistent with the clinical diagnosis of INO (Fig. 2). An additional region of restricted diffusion indicating an acute infarct was detected in the right inferior cerebellar hemisphere (Fig. 3). The presence of multiple brain infarcts in this patient supported the etiology of embolic stroke from the catheterization procedure. Magnetic resonance angiography revealed no significant cerebral artery disease (Fig. 4). The patient was treated in the stroke unit for 5 days according to stroke protocol, with medications including intravenous hydration of 1.5 mL/kg/hour for 2 days, dual antiplatelets, and statin, and 6 weeks after symptom onset, the patient achieved full neurological recovery. She received complete treatment for diffuse large B-cell lymphoma, which consisted of six cycles of the R-CEOP regimen to reduce the risk of further cardiac function deterioration and intrathecal methotrexate for high central nervous system (CNS) involvement risk, which was accompanied by clopidogrel withholding. She finally achieved a complete molecular response and remained uneventful after 6 months of treatment.

## Discussion and conclusions

We hereby present the case of a procedural stroke that occurred during diagnostic catheterization in a patient with hematologic malignancy who needed a clear diagnosis of the etiology of cardiomyopathy to proceed with chemotherapy treatment. Making the right choice requires precision medicine to differentiate this kind of stroke from other typical strokes, particularly in individuals with complex medical histories.

**Fig. 5:**
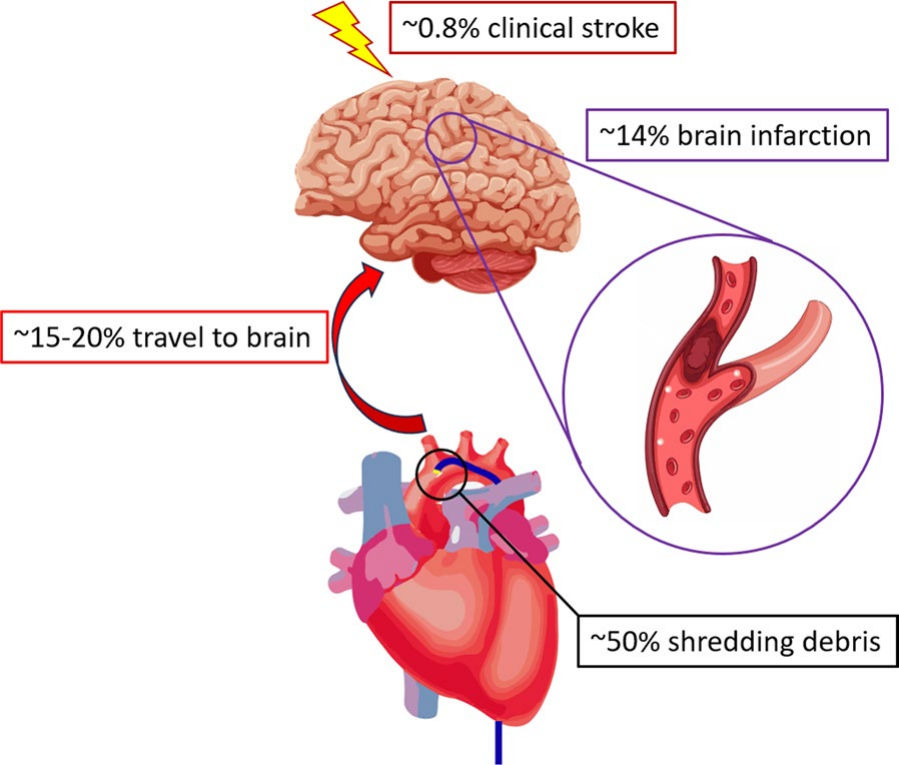
Probability of procedural stroke in different cascades

Procedural stroke has been hypothesized as resulting from atheromatous plaque embolism, dislodgement of device material, and air leakage, with varying levels of risk depending on the individual patient’s condition and the type of intervention. Interestingly, debris can be found in more than 50% of catheters used in percutaneous revascularization procedures [9]. Embolized debris must flow from the aorta into the brain circulation and lodge in a specific brain area to cause clinical stroke. The brain is generally assumed to receive 15–20% of the cardiac output [10]. When these data are taken into account as a theoretical risk, they are comparable to the findings of a meta-analysis of magnetic resonance imaging studies showing that 14% of patients undergoing percutaneous coronary intervention had silent brain infarctions [11]. In the present case, the risk ratio of focal neurologic deficit to silent brain infarction was only 0.06, which can be converted into an approximately 0.8% absolute risk of percutaneous intervention-related clinical stroke (Fig. 5). This level of risk is consistent with the overall rate of clinical stroke in another real-world study, which showed a risk of 0.18–0.44% for percutaneous coronary interventions and 0.06% for diagnostic coronary angiography [3].

Many risk factors contribute to procedural stroke. Atherosclerotic risk factors include advanced age, diabetes mellitus, arterial hypertension, and renal failure [12], all of which are unmodifiable or partially modifiable. Procedure-related risk factors include the type of procedure [11], access site [5], bulkiness of the catheter [13], catheter curvature [9], and contrast volume [13]; these are relatively more modifiable with pre-procedural planning and operator awareness.

INO is a rare presentation of stroke, especially procedural stroke. Occurrence of stroke at the medial longitudinal fasciculus results in ipsilateral adduction impairment and contralateral horizontal abduction nystagmus [8]. Although INO itself typically has a favorable prognosis [14], more than one brain infarct may be present in patients with procedural stroke, and this can affect the overall prognosis [15]. According to data from multiple large registries [3], procedural stroke is associated with substantial morbidity and mortality, with mortality rates ranging from 22–37%. Therefore, as for other types of strokes, an appropriate basic approach to stroke care remains critically important. This includes timely and accurate diagnosis, effective logistics for choosing an optimal revascularization technique, and availability of specialized stroke care units.

In conclusion, we have herein reported an unusual presentation of procedural stroke that is likely to be misdiagnosed, particularly by a medical care team inexperienced with INO. Despite the good prognosis of INO, appropriate stroke care remains indispensable in patients with procedural stroke because of the risk of multiple brain infarcts.

## Data Availability

The clinical data, imaging, and laboratory results in this case report are available from the corresponding author upon reasonable request. The patient’s privacy and confidentiality will be maintained in accordance with ethical guidelines and regulations.

## Abbreviation

INO: Internuclear ophthalmoplegia
